# Grave Robbing

**Published:** 1883-01-20

**Authors:** 


### *GRAVE ROBBING.*

With each succeeding winter a sensation is sprung at one or more points in regard to body-snatching. This time it comes from Philadelphia and Montreal.

Let the legislatures of the several States act wisely and pass laws giving to the colleges all unclaimed bodies and the bodies of any convicts and criminals not claimed, and we shall have no more grave robbing. The colleges are not inclined to draw anatomical material from what are called respectable sources, and know how to appreciate those natural and tender sensibilities which exist upon this subject. The newspapers are to be blamed for stirring up these sensations, and nine cases in ten they grow out of some indiscretion by parties who, perhaps, have been engaged and instructed to secure anatomical material, but to do so only from legitimate and proper sources.

